# Effects of intraperitoneal injection of magnetic graphene oxide on the improvement of acute liver injury induced by CCl_4_

**DOI:** 10.1186/s40824-020-00192-5

**Published:** 2020-08-26

**Authors:** Tahereh Foroutan, Fatemeh Ahmadi, Fariborze Moayer, Sahar Khalvati

**Affiliations:** 1 Department of Animal Biology, Faculty of Biological Sciences, Kharazmi University, Tehran, Iran; 2 Department of Pathobiology, School of Veterinary Medicine, Karaj Branch, Islamic Azad University, Karaj, Iran

**Keywords:** Magnetic graphene oxide, Liver, Nanomaterial

## Abstract

**Background:**

Liver failure is usually associated with the inflammation and oxidation of hepatocytes. Due to their unique properties, graphene and graphene-based nanostructures such as magnetic graphene oxide (MGO) are useful in biomedicine and engineering. In this study, synthesized MGO was used to improve the liver failure induced by carbon tetrachloride (CCl_4_). The hepatoprotective effects of intraperitoneal injection of MGO on the rat model of CCl_4_-induced acute liver failure were investigated.

**Materials and methods:**

In order to provide a rat model of acute liver failure, male rats were intraperitoneally injected with 2 ml/kg body weight CCl_4_. In the experimental groups, rats received 2 ml/kg CCl_4_ and 300 mg/kg MGO body weight simultaneously. Four days after injection, symptoms of acute liver failure appeared. The control, sham, CCl_4_, and CCl_4_ + MGO groups were compared and analyzed both histologically and biochemically.

**Results:**

The results indicated that the MGO injection reduced all CCl_4_-induced liver failure such as necrosis, fibrosis, inflammation, aspartate transaminase (AST), alanine aminotransferase (ALT), and alkaline phosphatase (ALP) in the experimental groups of the rat model of acute liver failure.

**Conclusion:**

The hepatoprotective effects of MGO might be due to histopathological suppression and inflammation inhibition in the liver.

## Background

The liver is the main detoxification and metabolic organ in the body. It is therefore vulnerable to different risk factors as well as chronic and acute failures [1]. Hepatic fibrosis is the wound healing response to chronic liver failure caused by viral infections, alcohol abuse, cholestasis, which is characterized by the accumulation of fibrillar extracellular matrix protein, etc. [1–3]. The membrane components of damaged hepatocytes and infiltrating inflammatory cells can activate Kupffer cells during liver fibrogenesis [4]. Their activation leads to the release of profibrotic factors such as the transforming growth factor, reactive oxygen species, hepatic stellate cells (HSC), and activates important collagen-producing cells in the liver [5, 6]. HSC activation is characterized by an enhancement in cell growth. In addition, the overproduction of extracellular matrix leads to liver fibrosis [7–9]. Inflammation can greatly stimulate hepatic fibrosis and HSC activation [5].

Liver disorders are associated with mortality risks, and over 100 million people suffer worldwide [3]. Acute hepatic failure (fulminant) as a dramatic clinical syndrome is caused by massive hepatic necrosis [10]. The induction of lipid peroxidation by active oxygen species plays a role in the pathogenesis of acute liver failure [9, 11]. Despite spectacular medical advances, there is no specific medication for stimulating liver function to protect it from failure, or regenerate liver cells [9, 12].

Today, in addition to regenerative medicine and laser therapy, the use of nano materials has also attracted a lot of attention in medical applications [13, 14]. Due to their unique properties, graphene and graphene-based nanostructures such as magnetic graphene oxide (MGO) are useful materials in biomedicine and engineering [15–17]. Graphene oxide (GO) as a new class of carbon-based materials is a derivative of graphene with a two-dimensional honeycomb structure. The major difference between the graphene and GO is the controllable hydrophilic nature of GO. The hydrophilic nature of GO is because of the existence of several hydroxyl groups on its surface, which makes it resistant to electron transfer. Due to its intrinsic optical properties as well as its small size, ease of use, and large specific surface area [18], GO is recommended for biomedical applications, including biosensors [5], drug/gene delivery [6, 7], and antibacterial effects [8]. GO has been reported to possess thermal, electrical, mechanical, and optical properties [19–22]. Biomaterials can be used to promote cell differentiation, attachment, and proliferation. For example, they are used for bone regeneration therapy with stem cells [23].

Growth factors and inducers are crucial for the proliferation and differentiation of stem cells [24–26]. The effectiveness of carbon nanostructures, as well as their modern two- and three-dimensional murid structures have been investigated for regenerative medicine. Graphene and its derivatives have been reported to improve the differentiation potential of stem cells into different lines based on the material types and stem cells [27–29]. These materials have been employed to deliver genes and growth factors into mesenchymal stem cells to manipulate their differentiation [30]. A new approach for the deposition of iron oxide nanoparticles on GO has been proposed, which leads to magnetic GO (MGO) and can be used to improve the biocompatibility of GO and Fe_3_O_4_ through chemical functionalization [29].

In this study CCl_4_ was used to induce acute liver failure. CCl_4_ is a well-known hepatotoxin, and is widely used to induce acute and chronic liver failures [9]. A three-dimensional nanoparticle was produced by taking advantage of the innovative capabilities GO and Fe_3_O_4_ nanoparticles in medical and therapeutic applications [29]. Possible in vivo hepatoprotective effects of MGO on the rat model of CCl_4_-induced acute liver failure were examined.

## Methods

Natural flake graphite powder was supplied by Qingdao Dingding Graphite Products (Shandong, Laixi, China). Other chemicals including H2SO4 98%, H2O2 30%, HCl 37%, and KMnO4 were obtained from Sigma-Aldrich Co. [23].

### Preparation of GO nanohybrid

GO was fabricated using modified Hummer’s method, which relied on the oxidation of graphite powder by a strong oxidant medium [31]. Graphite powder (0.5 g) was placed in a round bottom flask containing 50 mL of H_2_SO_4_ in an ice bath, and KMnO_4_ (2 g) was added gradually. The mixture was stirred for 2 h below 10 °C and kept for 1 h at 35 °C. Subsequently, the reaction medium was diluted with 50 mL distilled water in the ice bath, while the temperature was kept below 100 °C, and left to stir for 1 h. It further diluted to nearly 150 mL with distilled water. In order to eliminate excess permanganate ion, 10 mL of H_2_O_2_ 30% was added afterwards which turned the color of the reaction mixture into brilliant yellow. The final product was centrifuged and washed with 5% HCl followed by distilled water several times and the resulting solid was dried at 60 °C for 24 h. The preparation of MGO was based on the protocol by Kassaee et al. [32] and Foroutan et al. [33].

### Characterization

The prepared GO nanosheets were characterized using X-ray diffraction (XRD, Philips Xpert MPD Co. 1.78897 Å), scanning electron microscopy (SEM, Philips XL30 microscope, 25 kV accelerating voltage), transmission electron microscopy (TEM, Philips, EM208S, Netherlands, 100 kV acceleration voltage), atomic force microscopy (AFM, VEECO, CP-Research), and micro Raman spectroscopy (Almega Thermo Nicolet Dispersive Raman Spectrometer, excitation wavelength of 532 nm). MGO preparation was performed according to the previous study [32].

### Animals

The rats were kept in an animal room in a controlled temperature of 23 ± 2 °C and equal 12-h light/dark cycles with free access to food and water. All procedures were carried out in accordance with the Iranian code of conduct for the care and use of experimental animals for scientific purposes. The rat model of acute liver failure was prepared using a single intraperitoneal injection of CCl_4_ (2 ml/kg body weight) dissolved in sterile olive oil (1:1). After 4 days of injection, symptoms of acute liver failure such as biochemical and histological analysis were observed.

To investigate the effects of MGO on acute liver failure in the rat models, male rats were randomly divided into four groups: in the first group (control) rats were intraperitoneally injected with olive oil, in the second group (sham) they received 2 ml/kg body weight olive oil, in the third group (CCl_4_) they were injected with 2 ml/kg body weight CCl_4_, and in the fourth groups, they were injected with CCl_4_ and 300 mg/kg body weight MGO. The animals were anesthetized 24 h after the last injection using the mixture of ketamine 10% and xylazine 2% (both from Alfasan, Netherland). Blood samples from the heart were collected and the liver was then removed for histological examination.

### Histological tests

Liver tissues were fixed with formalin and embedded then in paraffin. Thin sections were stained with H&E, caspase-3 and IL-6. Hyperemia, apoptosis, and inflammatory cells were assessed according to no effect (−), mild effect (++), and intensive effect (+++). The Immunohistochemical (IHC) staining of caspase-3 and IL-6 was used to investigate the apoptosis and inflammation respectively. The number of apoptotic and inflammatory cells was also counted.

### Serum biochemical analysis

Blood samples were kept at room temperature for 1 h, and centrifuged then at 1500 g for 10 min at 4 °C. The serum was separated and kept in 20 °C before analysis. The activity of AST, ALT, and ALP was measured using an automated analyzer (Hitachi, Japan) and available kits (Pars Azmoon, Iran) according to the manufacturer’s instructions.

## Results

**Fig. 1:**
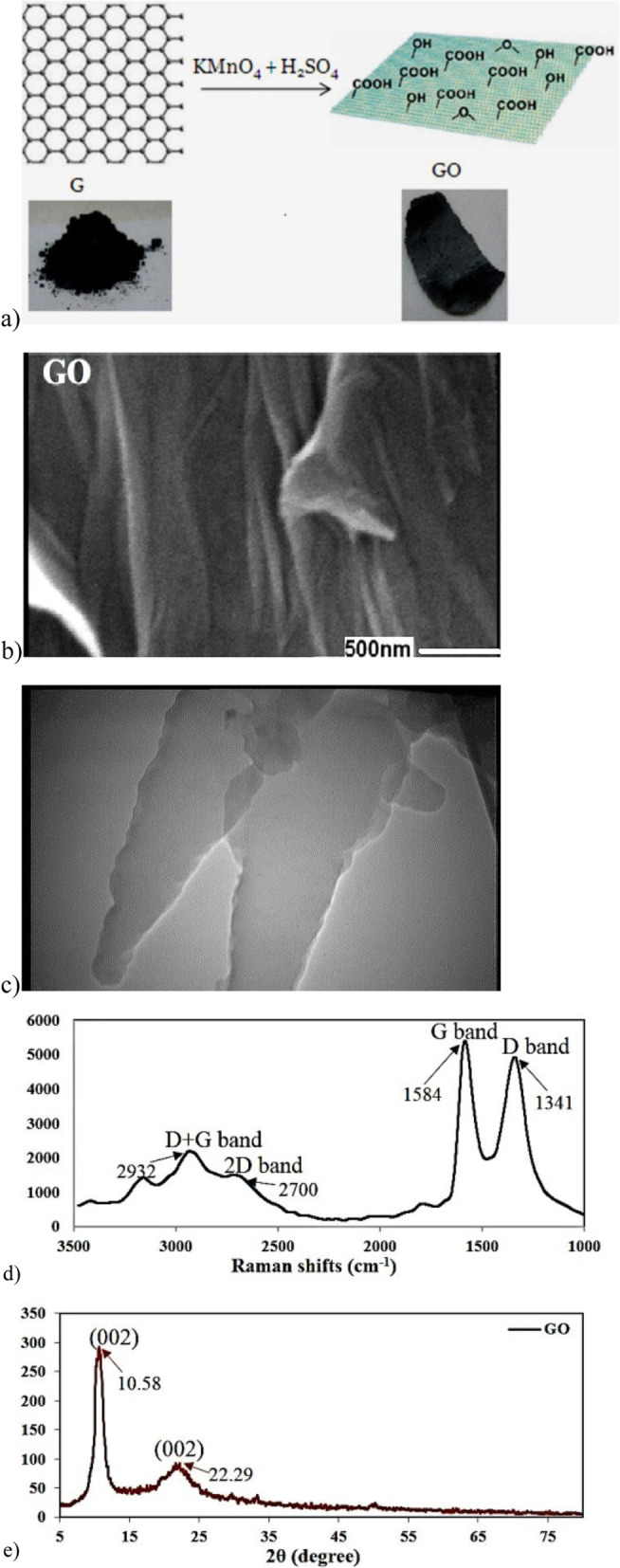
Synthessis of GO nanostructures from graphite (**a**); scaning electron microscopy (**b**); transmitision electon microscopy image of synthesized GO nanosheets (**c**); raman spectrum (**d**); XRD pattern of GO nanosheets (**e**)

**Fig. 2:**
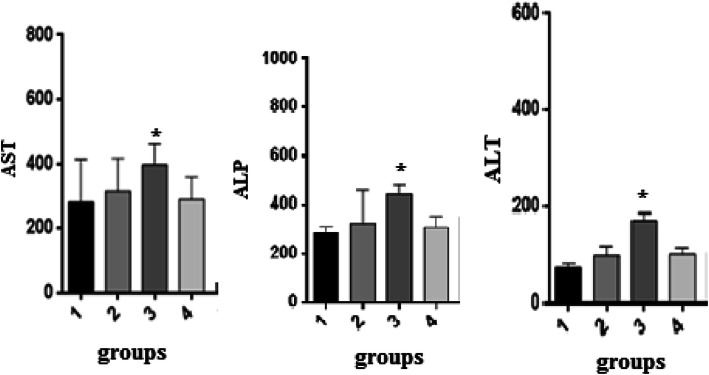
Effects of magnetic GO treatment on serum biochemical parameters in rat models of CCl_4_-induced acute liver failure. Magnetic GO treatment reduced serum ALT (**a**), AST (**b**), and ALP (**c**) in rats’ liver induced by CCl_4_ (*n* = 6). ALT: alanine aminotransferase, AST: aspartate, aminotransferase, ALP: alkaline phosphatase. *P* < 0.05. Control (1); sham (2); CCl_4_ (3); received 300 μg/kg weight body (4)

**Fig. 3:**
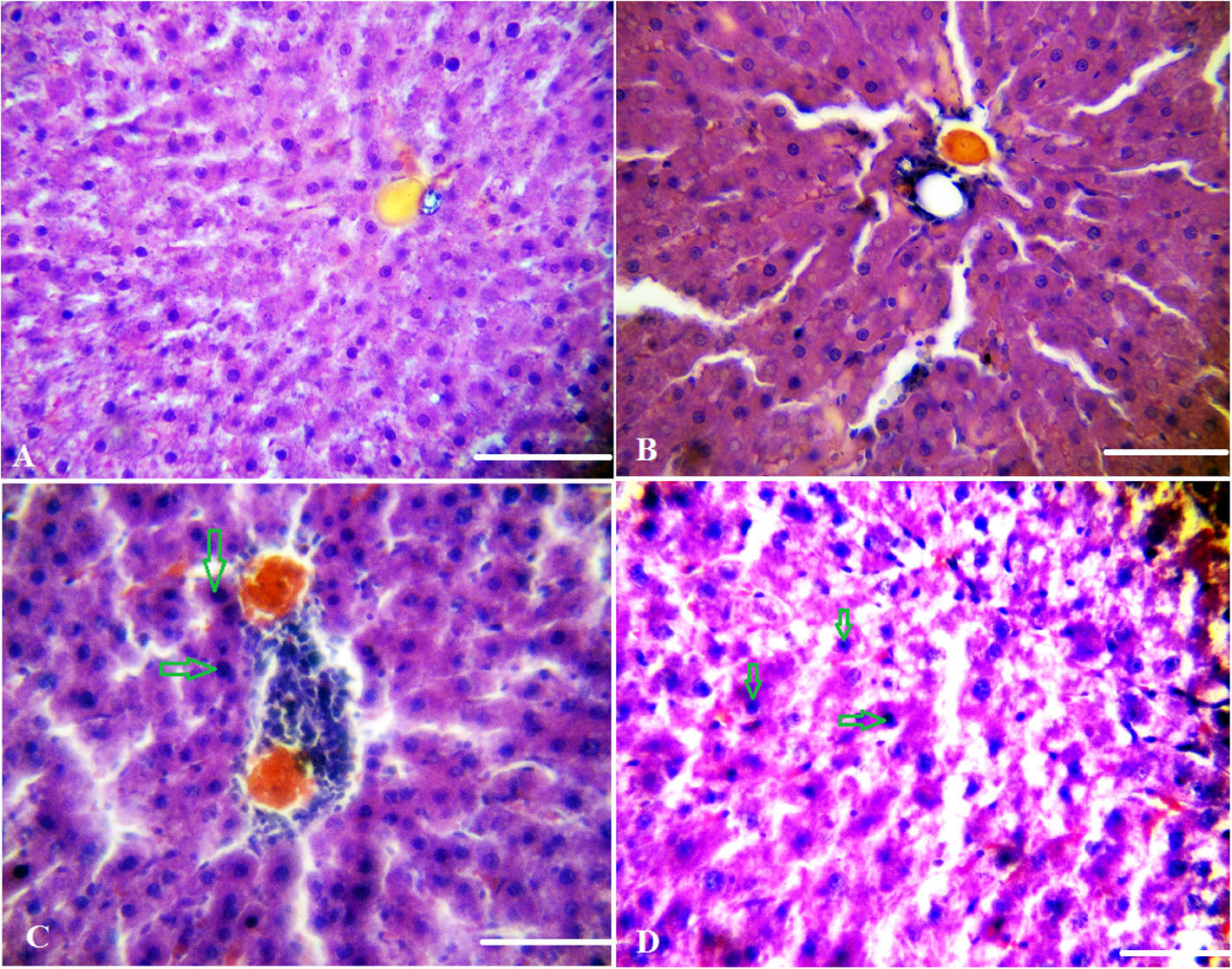
Photomicrograph of liver tissue in control (**a**), sham (**b**), and CCl_4_-induced liver failure (**d**, **c**) groups. **a** and **b** indicate normal liver cells. Arrows indicate apoptotic cells. H &E staining. Magnification: 400×. Scale bar = 100 μm

**Fig. 4:**
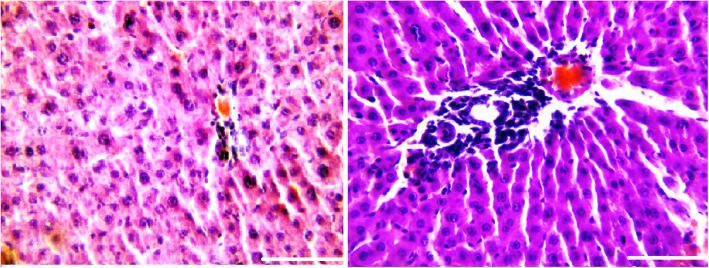
Effects of magnetic GO on the improvement of rat models of CCl_4_-induced acute liver failure. The reduction of dead cells and hyperemia in the experimental groups compared to the untreated group (Fig. 3c and d) is visible. Magnification: 400×. Scale bar = 100 μm

Figure 1 shows the schematic representation of synthesis of the GO nano-hybrid and also its characterization. AST, ALT, and ALP are the pathological indices for hepatic death and failure [1]. As compared to the CCl_4_ group, the ALT/AST/ALP levels were significantly reduced in the CCl_4_ + MGO group (*p* < 0.001) (Fig. 2). CCl_4_ administration caused hepatic failure, including hepatocyte apoptosis, inflammation, and hyperemia (Table 1). The protective effects of MGO were assessed through biochemical and histological examination of the samples. The number of inflammatory cells (neutrophils, lymphocytes, and Kupffer cells) was counted using a 400x magnification optical microscope in 10 visual fields in the H&E slides of each sample (Figs. 3, 4 and Table 1). The results of H&E staining showed that the CCl_4_ injection led to a significant apoptosis and inflammation of liver cells, while MGO significantly reduced the area of apoptosis as well as the inflammatory cells. Moreover, a significant increase in the inflammatory cells in the CCl_4_ group compared to the control group was observed. The results also suggested the effectiveness of MGO treatment in reducing inflammatory and apoptotic cells (*p* < 0.001). The number of dead cells and the incidence of hyperemia in the experimental groups were lower than those in the control group. Intraperitoneal injection of CCl_4_ induced apoptosis and inflammation in the liver cells. Our results showed that the administration of MGO significantly reduced the apoptotic and inflammatory liver cells induced by CCl_4_.

**Table 1:** Microscopic evaluation of hepatocytes in different treatments groups

Sample	Hyperemia	Accumulation of inflammatory cells	% apoptosis	Average apoptotic cells per field
1	–	–	0	–
2	–	–	0	–
3	+++	+++	17.71	52
4	++	++	11.14	29

1: Control group; 2: Sham group (olive oil); 3: CCl_4_ + olive oil; 4: CCl_4_ + olive oil + magnetic GO. no effect (−), mild effect (++), intensive effects (+++). Evaluation of the infiltration of inflammatory cell and hyperemia: slight (+); mild (++); intense (+++)

**Fig. 5:**
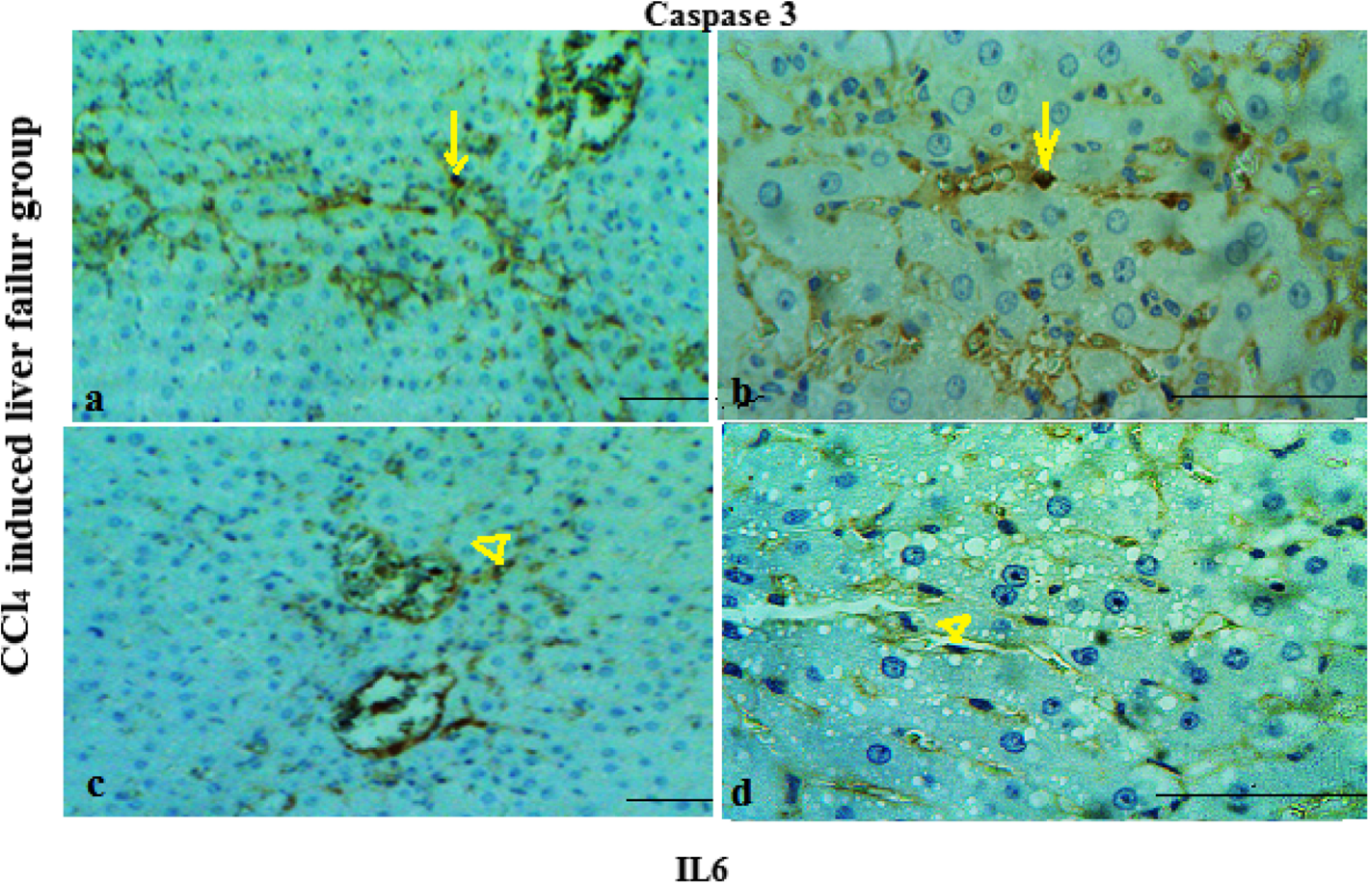
Micrographs of the CCl_4_-induced liver failure stained with caspase-3 (a, b) and IL-6 (c, d) antibodies. Arrows and head arrows indicate apoptosis and inflammatory cells respectively. Numerous positive caspase-3 and IL-6 are visible. Magnification: 100x (**a**, **c**) and 400× (**b**, **d**). Scale bar = 100 μm

**Fig. 6:**
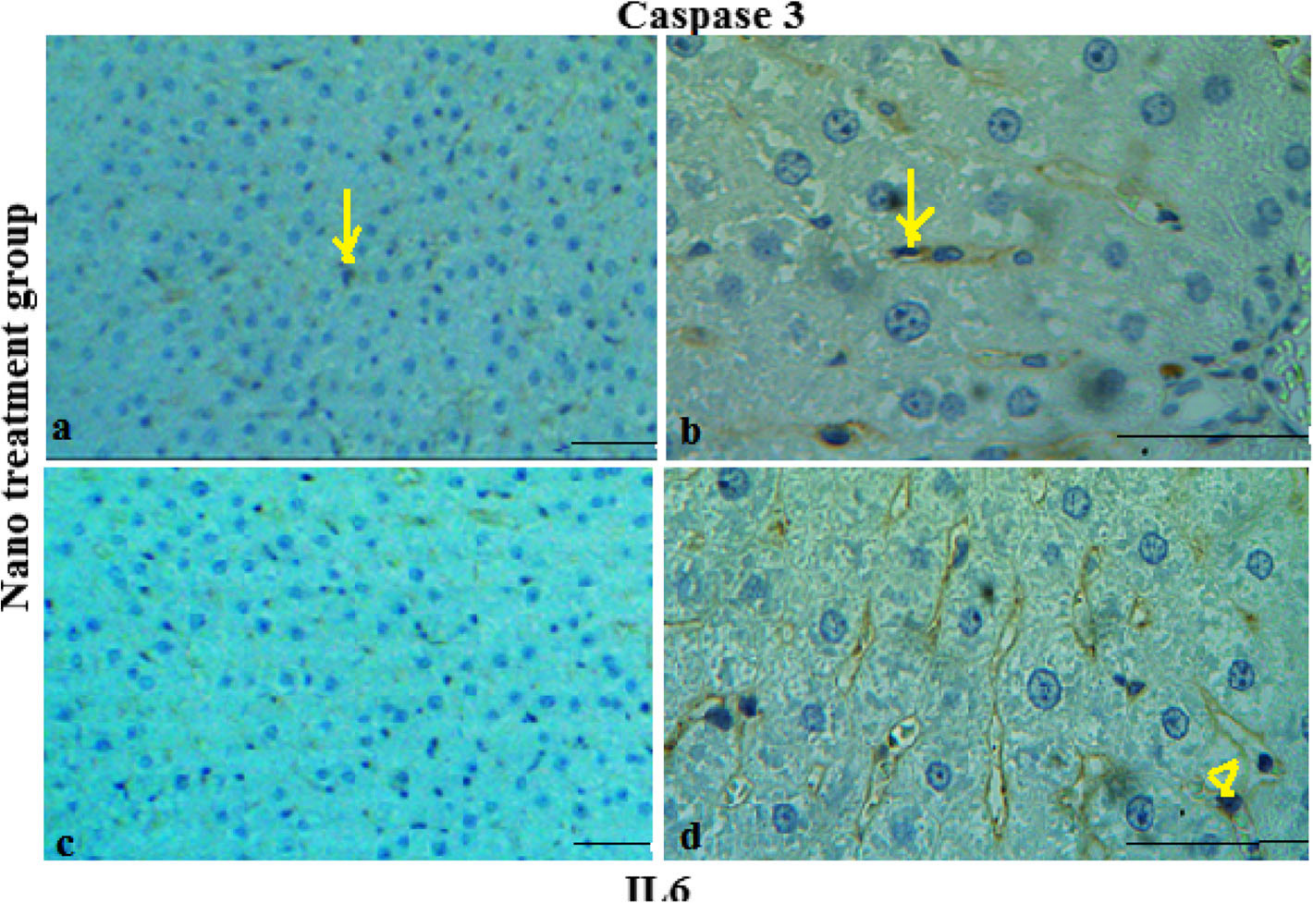
Micrographs of the CCl_4_-induced liver failure treated with nano-injection stained with caspase-3 (**a**, **b**) and IL-6 (**c**, **d**) antibodies. Arrows and head arrows indicate apoptosis and inflammatory cells respectively. Magnification: 100× (**a**, **c**) and 400× (**b**, **d**). Scale bar = 100 μm

**Fig. 7:**
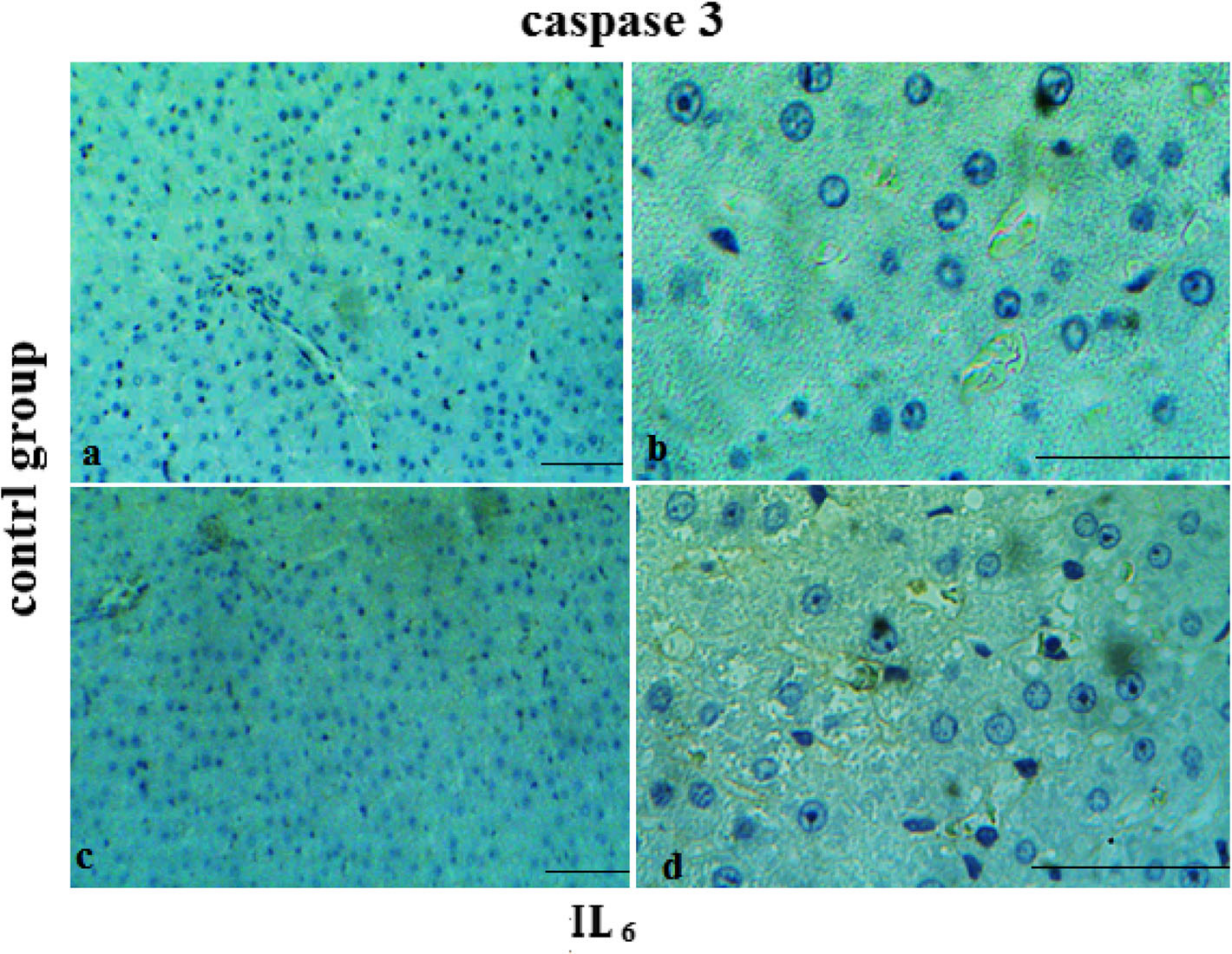
Micrographs of the liver in control group (received no treatment) stained with caspase-3 (**a**, **b**) and IL_6_ (**c**, **d**) antibodies. Magnification: 100x (**a**, **c**) and 400x (**b**, **d**)

The results of IHC staining showed that MGO plays an anti-apoptotic and anti- inflammation roles in the repair of damaged tissue. Positive nuclear staining for caspase3 and IL-6 markers occurredin greater than 20 and 25% of the liver cells of failure models respectively, which indicated a high apoptosis and inflammation index, compare to the MGO treatment (10%) and control (8%) groups respectively (Figs. 5, 6 and 7).

## Discussion

It has been shown that CCl_4_-induced hepatotoxicity can lead to acute liver failure with fibrosis, cirrhosis [9, 34], and hepatocellular necrosis [35]. Cytochrome P450 metabolizes CCl_4_ into free radicals trichloromethyl (CCl_3_) or trichloroperoxyl (CCl_3_O_3_) in the liver [36]. These free radicals cause lipid peroxidation which leads to hepatocytes necrosis, and induces inflammation. It further promotes the progress of hepatic fibrogenesis (Perez Tamayo, [37]), which in turn results in lipid peroxidation, hepatocytes necrosis and inflammation. Oxidative stress is closely associated with fibrosis and hepatic necrosis [38]. There is also a connection between the serum enzymes activities with liver parenchymal failure and the increased level of these enzymes can be used as a marker for the detection of acute liver damage [39]. The free radicals induced by CCl_4_ attack hepatocytes and cause parenchymal cell death. This in turn leads to inflammatory reactions in the liver [20]. The infiltration of inflammatory cells plays an important role in the progress of thymus damages.

Since deposition of iron oxide nanoparticles on GO has been suggested for improving the chemical functionalization of GO, in the present study we used the magnetic form of GO to treat liver failure [29]. The applicability of magnetic nanoparticles (tissue repair, drug delivery, biosensor technology) with tailored surface properties and appropriate physicochemistry has been investigated [40, 41]. The strong magnetic properties of Fe_3_O_4_ have gained particular attention for medical and biotechnological purposes [42–44]. The combination of GO with various polymers has applications in drug delivery [45]. Such limitations might cause inappropriate drug loading and rapid drug elution [46]. However, polymeric hydrogels suffer from limitations such as toxicity, unstable physiological conditions, inhomogeneous structure, and the presence of cross-linking agents [47].

In our study, we observed a significant reduction in AST and ALT serum levels (which are pathological indices for hepatic death and hepatic failure) in the CCl_4_ + MGO group compared to the CCl_4_ group (*p* < 0.001). Our previous study demonstrated the effectiveness of intraperitoneal injection of GO in improving the cisplatin-induced acute kidney failure [32]. On the other hand, the chemical functionalization of GO and Fe_3_O_4_ can be used to improve its biocompatibility [29]. Because of its low toxicity and strong magnetic properties, Fe_3_O_4_ has attracted great attention in medicine and biotechnology [29].

It has been reported that in the presence of GO, serum proteins and growth factors are more efficiently adsorbed on the surface of cells [46, 48]. The more Fe_3_O_4_@GO is adsorbed; it provides more essential biomolecules for the cell growth. A cell secretes various compounds for its growth and communication with surrounding cells. These substances are adsorbed on the surface of GO@Fe_3_O_4_ through ionic bonding and hydrophobic interactions, and affect cell proliferation and differentiation. Inflammatory cells can generate a broad range of cytokines, in particular IL_6_ and TNF-α [49]. High levels of IL_6_ can cause hepatic HSC activation and extra cellular matrix production, which promotes fibrosis in the liver. This study demonstrated that MGO significantly reduced infiltrating inflammatory cells in the liver after the CCl_4_ treatment. Due to the suitability of the product with its high cytocompatibility in biomedicine, for example as a drug carrier [39], we concluded that MGO accelerates the improvement of acute liver failure. The results for IHC staining showed that the MGO injection led to a significant reduction in the apoptotic (29 ± 0.2) and inflammatory cells (23 ± 0.3) in the CCl_4_-induced liver (*P* < 0.05) compared to liver induced by CCl_4_ group (52 ± 0.2) and (41 ± 0.3) respectively. Apoptosis is associated with various pathological situations, and occurs in response to a variety of cytotoxic stimuli. It plays a key role in developmental biology. Various mediators are secreted from immune system cells during the inflammation, such as IL_6_, which intensify the immune response [49]. IL_6_ has anti-inflammatory effects and leads to the releases of liver proteins in the acute stage. Indeed, inflammation and its symptoms are the result of a rapid increase in the secretion of inflammatory mediators such as IL-1 and IL_6_ [50, 51]. IHC staining confirmed that the use of MGO could reduce the number of apoptotic and inflammatory cells caused by liver failure.

## Conclusion

This study indicated that treatment with MGO helped improve CCl_4_-induced acute liver failure. MGO surface improved adsorption of secreted growth factors within the blood. It enabled hepatocytes to better reach and interact with damaged and healthy cells. Moreover, MGO enhanced cells’ interactions with each other as well as with extra cellular matrix. It appeared therefore that MGO could be used in combination with hepatocytes to treat diseases in vivo. We concluded that MGO reduces apoptosis and inflammation of liver cells and accelerates the absorption and loading of growth factors and therefore improved the protective effects of cells.

## Data Availability

All data generated and analyzed during the current study are available from the corresponding author on reasonable request.

## Abbreviations

MGO: Magnetic graphene oxide
CCl_4_: Carbon tetrachloride
AST: Aspartate transaminase
ALT: Alanine aminotransferase
ALP: Alkaline phosphatase
HSC: Hepatic stellate cells
GO: Graphene oxide
IHC: Immunohistochemical
IL_6_: Interleukin 6
H&E: Hematoxiline and eosin
